# On the Influence of Pleurisy in the Development of Phthisis

**Published:** 1864-11

**Authors:** 


### ON THE INFLUENCE OF PLEURISY IN THE DEVELOPMENT OF PHTHISIS—By Dr. Beau.

In the hospital of La Charité is a patient whose case, though apparently of little importance, has given to Dr. Beau the opportunity of pointing out the influence which pleurisy seems to exert on the development of phthisis. Between these diseases there is a very close connection; often pleurisy merely supervenes upon phthisis; but it is not uncommon to see a pleurisy occur in a subject who, till then, has presented no rational sign of phthisis, and to see it followed by the development of that disease. This was the opinion of Broussais, who attributed to inflammation the formation of tubercles, and Dr. Beau has met with many facts which have led him to the same conclusion. Thus, in the case of the patient in question nothing indicated a year ago that he was tubercular. This winter he took a pleurisy, perhaps two; when admitted into La Charité there was still a little effusion into the left side, which remained persistent, and was accompanied with a little febrile excitement towards evening. A blister removed the effusion, but did not lead to the disappearance of the fever; the patient was then carefully auscultated, and the presence of tubercle was recognized in the left inferior scapular region. Cases of the kind are not uncommon. Dr. Beau had for his house physician a young man who had contracted pleurisy; two years later he died tubercular. At this very time he has under treatment a patient whom two years ago he treated for pleurisy, and who is now tubercular. Many other examples could be quoted of tubercle supervening upon pleurisy. Is this any reason for treating these patients according to the system of Broussais? By no means; a very spare diet is bad because it debilitates, and an enfeebled state of the organism opens the door to all the diseases which afflict humanity.—*Edinb. Med. Journal*, August, 1864.—*Am. Jour. Med. Sciences.*

